# Traumatic pseudolipoma causing facial asymmetry: An uncommon pathology and review of its pathogenesis

**DOI:** 10.4103/0973-029X.80012

**Published:** 2011

**Authors:** Kunal Sah, Sunira Chandra, Alka Kale, Seema Awasthi, Priyanka Rastogi

**Affiliations:** *Department of Oral Pathology and Microbiology, VK KLE Institute of Dental Sciences, KLE University, Belgaum, Karnataka, India*; 1 *Department of Oral Medicine and Radiology, Teerthanker Mahaveer Dental College and Research Centre, Teerthanker Mahaveer University, Moradabad, Uttar Pradesh, India*; 2 *Department of Oral Pathology and Microbiology, VK KLE Institute of Dental Sciences, KLE University, Belgaum, Karnataka, India*; 3 *Department of Pathology, Teerthanker Mahaveer Dental College and Research Centre, Teerthanker Mahaveer University, Moradabad, Uttar Pradesh, India*

**Keywords:** Buccal fat pad, facial asymmetry, pseudolipoma, trauma

## Abstract

We present an uncommon case of traumatic pseudolipoma in a 24-year-old female, causing facial asymmetry. Literature review suggests trauma as a possible etiology for its pathogenesis, which was present in this case. Microscopically, sometimes it is difficult to differentiate between normal adipose tissue and lipoma. Clinician must provide accurate clinical information in order to make a definitive diagnosis of traumatic pseudolipoma. Its pathogenesis has also been highlighted in this article.

## INTRODUCTION

Lipomas are benign mesenchymal neoplasms composed of mature adipocytes, usually surrounded by a thin fibrous capsule.[1] They are the most common soft tissue tumors, and about 20% of cases occur in the head and neck region. However, only 1-4% of cases involve the oral cavity. Oral lipoma represents 0.5-5% of all benign oral cavity neoplasms and usually presents as painless, well-circumscribed, slow-growing submucosal or superficial lesions, mainly located in the buccal mucosa.[1–4]

The term traumatic pseudolipoma was proposed by Brooke and MacGregor. Some speculate, trauma serves as a cause of fat necrosis, may trigger the formation of lipoma “like”, so called “traumatic pseudolipoma.” Several reports of similar cases, all subsequent to trauma, gave evidence for the acceptance of the term traumatic pseudolipoma.[5–10]

## CASE REPORT

A 24-year-old female reported with a chief complaint of asymptomatic swelling of the left side of the face, since 4 years. She gave history of trauma 4 years back in the same region, due to pressure cooker burst. Since then, the swelling had reduced to the present size and is constant since 1 year.

**Figure 1:**
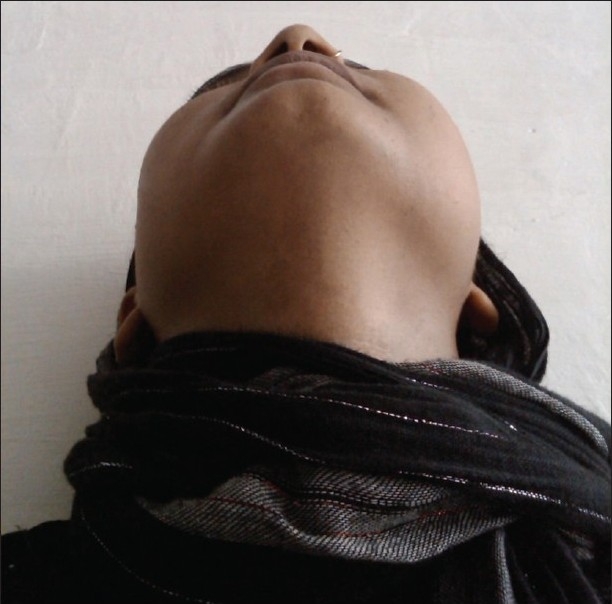
Clinical photograph of the patient showing facial asymmetry on the left side

Extraoral examination revealed a swelling, measuring 3 × 3 cm in diameter, extending superiorly from inferior orbital process, mesially up to ala of the nose, distally to zygomatic process and approximately 3 cm inferiorly [Figure 1]. On palpation, a deep-seated soft tissue mass was felt. It was nontender, mobile and soft in consistency. Overlying skin of the patient was normal. Intraoral examination revealed no significant findings. Posterioanterior radiographic view was taken and was insignificant.

**Figure 2:**
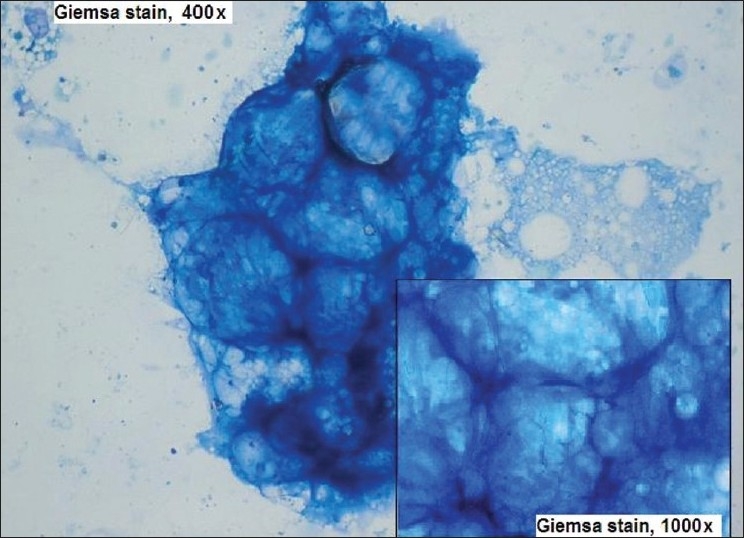
FNA smear displaying vacuolated cells with nucleus at the periphery suggestive of mature adipocytes (Giemsa stain, 40×, 1000×)

As the mass was deep seated (the tumor was located subcutaneously, but superficial to the musculofascial system), fine needle aspiration (FNA) cytology was done. Aspiration consisted of jelly-like colorless material. Slides were prepared, air dried and stained with Giemsa stain. On cytopathological examination, the smear showed few areas of cellular material composed of vacuolated cell. Few of these cells showed nucleus at the periphery, which was suggestive of mature adipocytes [Figure 2].

**Figure 3:**
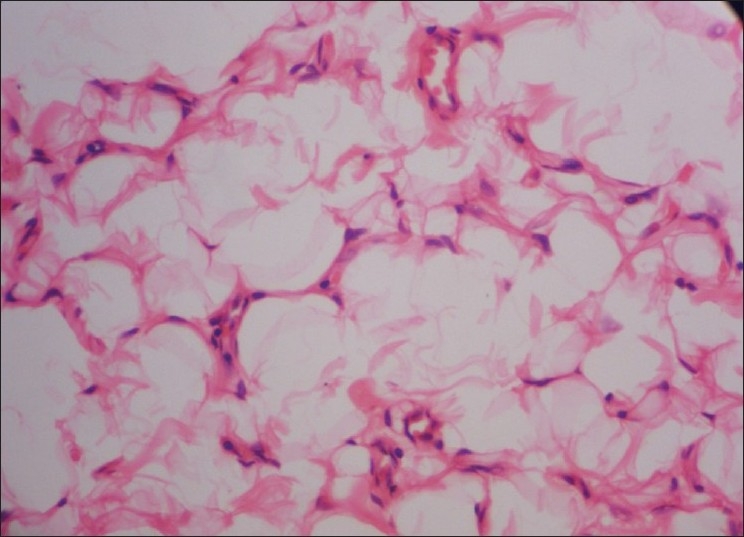
Photomicrograph showing sheets of mature adipocytes containing large clear cytoplasm with eccentric nuclei (H and E, 40×)

Surgical excision with facial recountering was done under general anesthesia via naso-endotracheal intubation. Microscopic examination of the excised soft tissue mass revealed sheets of mature adipocytes containing large clear cytoplasm with eccentric nuclei. There was no evidence of cellular atypia or metaplasia [Figure 3]. The tumor was not surrounded by the connective tissue capsule. These features are consistent with a classical diagnosis of a pseudolipoma. With clinical correlation and the history of trauma, a diagnosis of “traumatic pseudolipoma” was made.

## DISCUSSION

Pseudolipoma of the buccal fat pad into the oral cavity was first reported in literature by Clawson in 1968.[6] The buccal fat pad is an encapsulated mass of adipose tissue located between the buccinator and masseter muscles. Anteriorly, it extends mesial to the masseter muscle. It was suggested that this pad of fat may be a possible source of confusion in the diagnosis of lipoma.[11] It was first described in 1802 by Bichat, as being truely fatty in nature. In non-English speaking countries, it is commonly referred as the “Bichat’s ball”.[12]

This disease event typically occurs in children ranging from 4 months to 4 years of age when the fat pad is still quite large in proportion to the size of the face. The etiology of traumatic pseudolipoma of the buccal fat pad is speculated in infants and young children because of their frequent chances of falling down with an instrument such as a toothbrush in their mouth, and getting an oral cavity injury. Furthermore, negative intraoral pressure induced by their suckling activity may promote herniation of the buccal fat pad from the wound.[6–10,13,14]

The origin of these tumors, whether hamartoma or neoplasm is subject to question.[15] Brooke and MacGreggor have named this phenomenon “post-traumatic pseudolipoma”, owing to the absence of a true capsule.[5]

Adair *et al*.[16] were the first to report the role of trauma in the formation of lipoma, but few reports have attempted to explain a mechanism. Initial theories were purely mechanical: normal deep fat tissue prolapsing through Scarpa’s fascia, ruptures in the normal septa surrounding adipose tissue and scar contractures secondary to shearing facial injury.[5,17,18] Penoff[19] was the first to question the mechanical theory.

In a retrospective review of 31 cases with benign adipose tissue tumors, Aust *et al*. concluded that the existence of a pathogenic link between blunt soft tissue trauma and the formation of post-traumatic lipomas is still controversial. According to them, two potential mechanisms can occur. Firstly, the formation of so-called post-traumatic “pseudolipomas” may result from a prolapse of adipose tissue through fascia induced by direct impact. Secondly, it may occur as a result of pre-adipocyte differentiation and proliferation mediated by cytokine release following soft tissue damage after blunt trauma and hematoma formation.[20] Signorini and Campiglio[21,22] also discussed the possibility of pre-adipocyte differentiation to adipocytes by trauma due to cytokine release and hematoma formation.

Copcu and Sivrioglu[15] have described 10 cases of post-traumatic lipoma and have speculated that the formation of a post-traumatic lipoma can only occur with fat necrosis as a trigger, setting off a cascade of local inflammation to affect adipocytes and promote lipoma formation. Other reported cases have included buccal fat pad herniation into the oral cavity after blunt facial trauma and iatrogenic injury secondary to dental surgery.[14,22,23]

Recently, a genetic defect with translocation or partial loss of chromosome 12 has also been suggested as a factor contributing to the formation of lipomas. In addition, chronic local compression causing anatomical defects in the fascia may also result in pseudolipoma formation. Herbert and DeGeus reported a patient with an abdominal lipoma induced by wearing tight trousers. An extensive study on the origin of post-traumatic lipomas postulated that subcutaneous scar formation and contracture after a soft tissue trauma may also lead to the development of pseudolipomas.[24] Recently, Aust *et al*. found a link between elevated coagulation tests [elevated prothrombin time (PTT)] and the development of post-traumatic lipomas.[20] In the present case, trauma due to pressure cooker burst seems to play an important role in its development.

Histologically, it is indistinguishable from normal fat or *de novo* lipoma, and is simply described as normal fat in an abnormal location. Cells of the lipoma differ metabolically from normal fat cells. Furthermore, Fatty acid precursors are incoporated at a more rapid rate into the lipoma than into normal fat while lipoprotein lipase activity is reduced.[1]

## CONCLUSION

We present a case of subcutaneous fibrofatty lesion. It presented as a swelling in the cheek region causing facial disfigurement, as a result of trauma. Though aspiration cytology has little value in the diagnosis of superficial lipoma, it may be helpful in elucidating the nature of deep-seated tumor or tumor in unusual location. Clinical information provided by the clinician and its histopathological features helped in making a definitive diagnosis of “traumatic pseudolipoma”. Further clinical and experimental studies are necessary to clarify the pathogenesis and the mechanism of post-traumatic lipoma formation.
